# Photosystem Disorder Could be the Key Cause for the Formation of Albino Leaf Phenotype in Pecan

**DOI:** 10.3390/ijms21176137

**Published:** 2020-08-26

**Authors:** Ji-Yu Zhang, Tao Wang, Zhan-Hui Jia, Zhong-Ren Guo, Yong-Zhi Liu, Gang Wang

**Affiliations:** Institute of Botany, Jiangsu Province and Chinese Academy of Sciences, Nanjing 210014, China; maxzhangjy@163.com (J.-Y.Z.); immmorer@163.com (T.W.); 13915954315@163.com (Z.-H.J.); zhongrenguo@cnbg.net (Z.-R.G.); liuyz1965@163.com (Y.-Z.L.)

**Keywords:** pecan, albino leaves, differentially expressed genes, chlorophyll, photosynthetic systems, transcriptome analysis

## Abstract

Pecan is one of the most famous nut species in the world. The phenotype of mutants with albino leaves was found in the process of seeding pecan, providing ideal material for the study of the molecular mechanisms leading to the chlorina phenotype in plants. Both chlorophyll a and chlorophyll b contents in albino leaves (ALs) were significantly lower than those in green leaves (GLs). A total of 5171 differentially expression genes (DEGs) were identified in the comparison of ALs vs. GLs using high-throughput transcriptome sequencing; 2216 DEGs (42.85%) were upregulated and 2955 DEGs (57.15%) were downregulated. The expressions of genes related to chlorophyll biosynthesis (*HEMA1*, encoding glutamyl-tRNA reductase; *ChlH*, encoding Mg-protoporphyrin IX chelatase (Mg-chelatase) H subunit; *CRD*, encoding Mg-protoporphyrin IX monomethylester cyclase; *POR*, encoding protochlorophyllide reductase) in ALs were significantly lower than those in GLs. However, the expressions of genes related to chlorophyll degradation (*PAO*, encoding pheophorbide a oxygenase) in ALs were significantly higher than those in GLs, indicating that disturbance of chlorophyll a biosynthesis and intensification of chlorophyll degradation lead to the absence of chlorophyll in ALs of pecan. A total of 72 DEGs associated with photosynthesis pathway were identified in ALs compared to GLs, including photosystem I (15), photosystem II (19), cytochrome b6-f complex (3), photosynthetic electron transport (6), F-type ATPase (7), and photosynthesis-antenna proteins (22). Moreover, almost all the genes (68) mapped in the photosynthesis pathway showed decreased expression in ALs compared to GLs, declaring that the photosynthetic system embedded within the thylakoid membrane of chloroplast was disturbed in ALs of pecan. This study provides a theoretical basis for elucidating the molecular mechanism underlying the phenotype of chlorina seedlings of pecan.

## 1. Introduction

Plants synthesize the carbohydrates and energy needed for growth and development through photosynthesis in leaves. Leaf color directly affects photosynthesis. Usually, the leaf color is green; however, leaf color variations, including chlorina, albino, red, and green leaves, with white or yellow interion, have been observed in plenty of plants, such as tea plant [1,2,3], *Anthurium andraeanum* [4], red maple [5], and oilseed rapa [6]. To our knowledge, the occurrence of leaf color variations is a very complex biological process and is largely determined by genetic and environmental factors. Mutants with leaf color variations are ideal genetic material for exploring the physiological, biochemical, and molecular mechanisms of chlorophyll biosynthesis, chloroplast structure and function, and photosynthesis. Dismembering the leaf color variations’ fundamental mechanism is all-important for broadening the theoretical knowledge of plant growth and development.

The key aspects influencing leaf color formation are the contents of chlorophyll, carotenoid, and anthocyanin. Chlorophyll, which is synthesized in chloroplasts, is the major component in typical green leaves. The chloroplast is composed of the chloroplast membrane, thylakoid, and matrix. Chloroplast development, the number and size of chloroplasts, and chlorophyll biosynthesis in the leaf directly affect its color. Therefore, any fault in these processes can lead to the leaf losing the green color. Previous reports have shown that the expression of genes related to chloroplast development and chlorophyll biosynthesis can result in variations in leaf color. Those genes directly or indirectly regulate the structure of chloroplasts, chlorophyll biosynthesis, and several metabolic processes that affect the depth of leaf color have been identified in leaf color mutants [1,4,7,8,9]. Moreover, the variations of leaf color are affected by environmental factors, including light and temperature. Thus, it is very difficult to declare the leaf color variation mechanism due to the variations in leaf color being affected by complex environmental factors.

The pecan (*Carya illinoensis*), native to North America, is one of the most important economic nut trees in the world. Since the 21st century, the pecan industry has been rapidly developed in China. Some albino leaf seedlings were found from the pecan seed seedlings, which provided new ideal material for studying the molecular mechanism of leaf color formation. The mechanism of leaf color formation in pecan has not been investigated in detail so far. The pecan whole genome has been sequenced and reported [10,11], providing abundant genetic information for research on the transcriptome. Thus, the chlorophyll and carotenoid contents of the albino leaf (AL) and green leaf (GL) were measured in this study. Leaf transcriptomes from GL and AL were sequenced and differentially expressed genes between GL and AL were identified to fully understand the gene expression difference between AL and GL and explore the cause of albino leaf formation in pecan. This study will broaden our understanding of phenotypes in the leaf color variants. The results provide an appreciated resource for genetic and genomic studies in plants for leaf color formation.

## 2. Results

### 2.1. Content of Chlorophyll and Carotenoid in Green and Albino Leaves in Pecan

**Figure 1:**
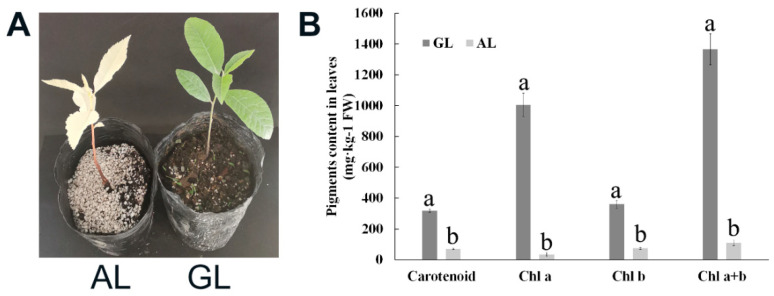
Chlorophyll content in leaves of pecan. (**A**): Photographs of green leaf (GL) seedling and albino leaf (AL) seedling. (**B**): Chlorophyll content in GL and AL. Mean values (±SD) of three biological replicates are shown. Different lowercase letters above the error bars indicate a significant difference of correlation at 0.05 levels (one-way ANOVA, *p*-value < 0.05).

A few pecan seedlings with albino leaves were found during the progress of seeding (Figure S1). It is well known that chlorophyll biosynthesis leads to leaf greening, and the chlorophyll contents of leaves from green leaf (GL) seedlings and albino leaf (AL) seedlings were measured, respectively (Figure 1). The results showed that in AL, both chlorophyll a and chlorophyll b contents were significantly lower than those in GL (approximately 3.46% and 20.87% of the contents in GL, respectively; Figure 1B). The ratio of chlorophyll a/b in GL was significantly lower than that in AL (Table S1). The carotenoid contents in AL were significantly lower than those in GL (Figure 1B), and the ratio of carotenoid/chlorophyll in AL was significantly high than that in GL (Table S1). These results suggested that albino leaves result from reduced chlorophyll levels and that the lower chlorophyll content might have resulted from abnormal chlorophyll biosynthesis and degradation.

### 2.2. RNA Sequencing of Leaf Transcriptomes of the GL and AL Seedlings and Mapping of RNA Sequences to the Reference Genome

RNA-seq, followed by strict quality control and processing, generated a total of 32.35 GB of clean data from 6 transcriptome libraries. The six transcriptome libraries represented two groups with three repetitions. After filtering out duplicate sequences and ambiguous and low-quality reads, we obtained a total of 231, 590, and 820 high-quality (HQ) clean reads: 115, 546, and 190 reads and 116, 044, and 630 reads were generated for GL and AL, respectively (Table S2). The average GC percentage was 45.51%, with a QC30 base percentage above 90.01%. Details on data and data quality, before and after filtering, are shown in Table S3. HQ clean reads were mapped to the pecan reference genome (Cil.genome.fa). Approximately 35.83 million clean reads (92.81% of the total) were mapped; 34.91 million were unique. An overview and detailed data are given in Table 1 and Table S2.

### 2.3. Differentially Expressed Gene Analysis

Three biological replicates were used for RNA-seq. To test sample repeatability, we calculated the correlation coefficient between the samples. The correlation coefficient in the repeat group was greater than 0.9375 (Figure S2), indicating the consistency among the three biological replicates. Thus, the RNA-seq results were confirmed to be highly reliable for further analyses.

**Figure 2:**
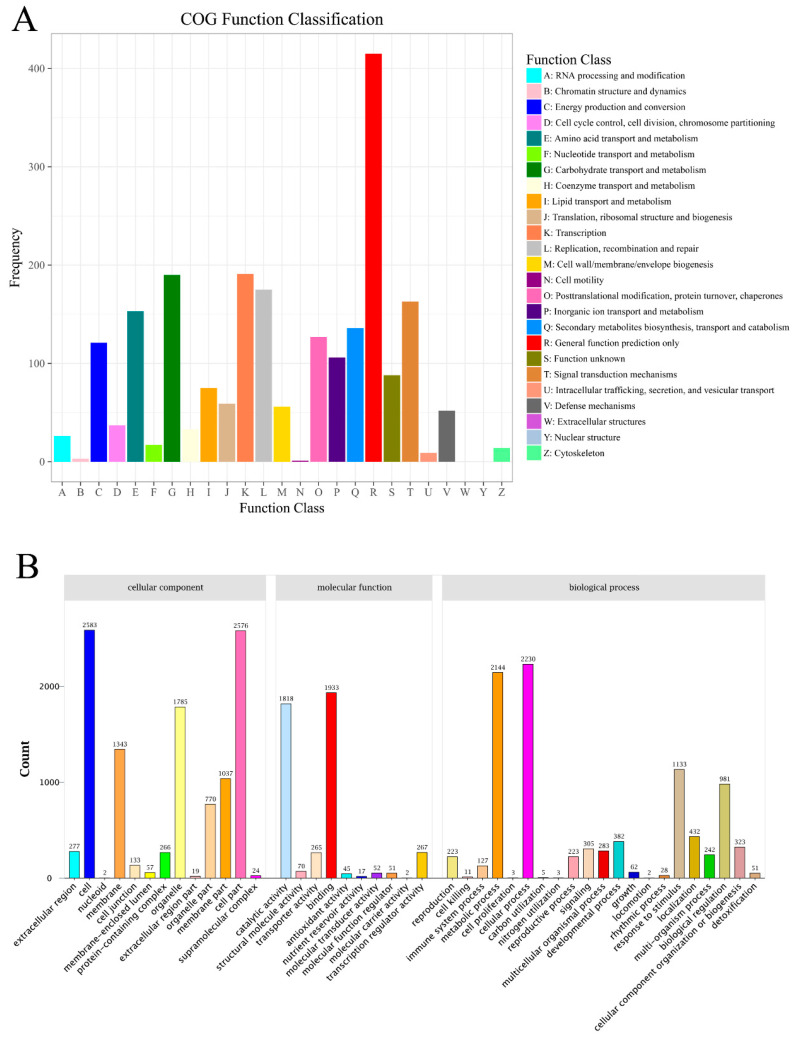
Functional annotation of differentially expressed genes of pecan in the comparison of AL vs. GL. (**A**): COG classification. (**B**): GO classification.

In the current study, a total of 5171 DEGs was identified in the comparison of AL vs. GL; 2216 DEGs (42.85%) were upregulated, and 2955 DEGs (57.15%) were downregulated (Table S3). Additionally, 4389 out of the 5171 DEGs (84.88%) were aligned to known proteins in the nr database, whereas 3596 (69.54%) could be annotated based on sequences in the Swiss-Prot database (Table 2 and Table S4). Moreover, 1546 (29.90%) DEGs were categorized in 25 cluster of orthologous groups of proteins (COG) (Figure 2A and Table 2). The three largest categories were (1) general function prediction only (415, 26.84%), (2) transcription (191, 12.35%), and (3) carbohydrate transport and metabolism (190, 12.29%).

In total, 3337 DEGs (64.53%) were categorized into three different GO trees of cellular components, molecular functions, and biological processes (Figure 2B, Table 2 and Table S5). The three main categories were further classified into 51 functional groups. In the category of cellular components, the largest groups were cell, cell part, and organelle. Binding, catalytic activity, and transcription regulator activity were the dominant groups in the molecular function category, and for the biological processes, DEGs with cellular process, metabolic process, and response to stimulus formed the major groups. The top-ten enrichment of GO were chloroplast thylakoid membrane, photosystem I, photosystem II, chlorophyll binding, reductive pentose–phosphate cycle photosynthesis, light-harvesting in photosynthesis, pigment binding, chloroplast envelope, photosynthesis, and integral component of membrane (Table S6). Furthermore, in order to understand the biological function of these DEGs, all DEGs were also mapped to terms in the KEGG database. Finally, 939 (18.16%) DEGs were matched and assigned to 128 KEGG pathways (Table S7). The first three biological pathways involved in photosynthesis (51), photosynthesis-antenna proteins (22), and metabolic pathways (360) were significantly enriched between AL and GL1 (Table 3).

### 2.4. Chlorophyll Metabolism-Related Genes Expression Analysis

To validate the RNA sequencing data, chlorophyll metabolism-related genes were selected for qRT-PCR analysis. The qRT-PCR results indicated that all of these DEGs exhibited similar expression kinetics to those obtained from the RNA sequencing analysis (Figure S3), thus supporting the validity of the method used for determining DEGs from the RNA sequencing analysis.

**Figure 3:**
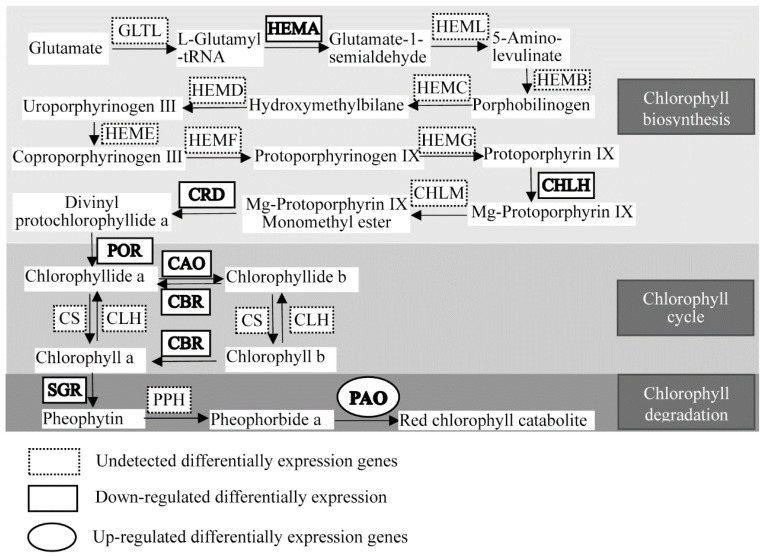
Chlorophyll metabolic pathway (KO00860) in albino leaves of pecan. CAO: chlorophyllide a oxygenase; CBR: chlorophyll(ide) b reductase NYC1; CHLH: magnesium chelatase H subunit; CHLM: Mg-proto IX methyltransferase; CLH: chlorophyllase; CRD1: Mg-protophyrin IX monomethylester (oxidative) cyclase; CS: chlorophyll synthase; GLTL: glutamate tRNA ligase; HEMA: glutamyl-tRNA reductase; HEMB: 5-aminolevulinate dehydrogenase; HEMC: porphobilinogen deaminase; HEMD: uroporphyrinogen III synthase; HEME: uroporphyrinogen III decarboxylase; HEMF: coproporphyrinogen III oxidase; HEMG: protoporphyrinogen oxidase; HEML: glutamate-1-semialdehyde; PAO: pheophorbide a oxygenase; POR: protochlorophyllide reductase; PPH: pheophytin pheophorbide hydrolase; SGR: STAY-GREEN LIKE.

Twelve genes involved in chlorophyll metabolism, including biosynthesis, cycle, and degradation, were expressed differentially in the comparison of AL vs. GL using de novo transcriptome sequencing (Table 4 and Figure 3). In chlorophyll biosynthesis, *HEMA1* (encoding glutamyl-tRNA reductase), *ChlH* (encoding Mg-protoporphyrin IX chelatase (Mg-chelatase) H subunit), *CRD* (encoding Mg-protoporphyrin IX monomethylester cyclase), and *POR* (encoding protochlorophyllide reductase) showed significantly lower expression in ALs than in GLs, indicating that chlorophyll biosynthesis was downregulated in ALs. Among genes related to the chlorophyll cycle, the expression of two *CAO* (encoding chlorophyllide a oxygenase) and three *CBR* (encoding chlorophyll (ide) b reductase NYC1) genes were also significantly lower in expression in ALs than in GLs. Among DEGs related to chlorophyll degradation, the expression of *SGR* (STAY-GREEN, encoding Mg-dechelatase) in ALs were significantly lower than those in GLs. However, two *PAO* (encoding pheophorbide a oxygenase) genes in ALs were significantly higher than those in GLs, indicated that chlorophyll degradation was upregulated in ALs.

### 2.5. Identified Differentially Expressed Genes Involved in Photosynthesis

A total of 72 DEGs associated with the photosynthesis pathway was identified in AL compared to GL (Table 5), including PSI (15), PSII (19), cytochrome b6-f complex (3), photosynthetic electron transport (6), F-type ATPase (7), and photosynthesis-antenna proteins (22). Moreover, almost all the genes (68) mapped in the photosynthesis pathway showed decreased expression in AL compared to GL except for *PsaC* (PSI, MSTRG.1756), *petH* (photosynthetic electron transport, CIL1219S0022), *A* (F-type ATPase, CIL0936S0006), and *B* (F-type ATPase, MSTRG.3669). The expressions of *PsaA*, *PsaD*, *PsaE* (2), *PsaF*, *PsaG*, *PsaH* (2), *PsaK* (2), *PsaL*, *PsaN* (2), and *PsaO* in PSI were downregulated in AL compared to GL; however, only the *PsaC* expression was upregulated. All of the DEGs in PSII were downregulated, including *PsbA*, *PsbB* (2), *PsbK* (2), *PsbO*, *PsbP* (3) *PsbQ* (3), *PsbR*, *PsbS* (2), *PsbW* (2), *PsbY*, and *Psb27*-*H1*. A *petB* and 2 *petCs* related to the cytochrome b6-f complex revealed a significant reduction in their expression levels. Additionally, 6 genes involved in the photosynthetic electron transport unveiled that *petEs* (CIL0131S0022 and CIL1192S0070), *petF*, petH, and petJ were downregulated and only one transcript of *petH* (CIL1219S0022) was upregulated when compared with control. Among the F-type ATPase-related DEGs, the expressions of 5 genes (*atpB*, *atpC*, *atpD*, *atpH*, and *atpB*) were downregulated and 2 genes (*A* and *B*) were upregulated in AL compared to GL. In addition, all of the photosynthesis-antenna protein-related genes (22) were found to be significantly downregulated in AL compared to GL, including *LHCA1*(2), *LHCA2*(2), *LHCA3*, *LHCA4*, *LHCA5*, *LHCA6*, *LHCB1*(4), *LHCB2*, *LHCB3*(2), *LHCB4*(2), *LHCB5*(3), and *LHCB6*(2) (Table 5). The results show that these genes may be associated with the leaf color variation and that the photosynthesis pathway was destroyed in ALs of pecan.

### 2.6. Response of Transcription Factors in the Comparison of AL vs. GL

Differentially expressed transcription factor genes were analyzed to identify the transcription factors involved in the regulation of chlorophyll metabolism in pecan (Table 6 and Table S8). Forty-two categories of different transcription factor families were identified in the comparison of AL and GL in this study (Table 2 and Table S8). We identified 40 MYB transcription factors expressed differentially and significantly, including 16 upregulated and 24 downregulated members, suggesting that MYB transcription factors could be involved in chlorophyll metabolism. Among the AP2/ERF transcription factor family, 23 members were upregulated and 12 members were downregulated in AL compared with GL. NAC, C2C2, C2H2, bHLH, and WRKY transcription factor families were over-represented in the list of regulated genes, indicating that those transcription factor families probably also play key roles in the transcriptional regulation of genes in the chlorophyll metabolism of pecan.

## 3. Discussions

A few pecan seedlings with albino leaves were found during the progress of seeding (Figure S1). Chlorophyll content was significantly lower than that in GL (Figure 1B), suggesting that the albino leaves resulted from reduced chlorophyll levels. In order to elucidate the key factors in the formation of AL mutation of pecan, de novo transcriptome sequencing and comparative analysis of DEGs were performed in comparing AL vs. GL. GO classification showed that genes associated with the chloroplast thylakoid membrane, photosystem I, photosystem II, chlorophyll binding, reductive pentose–phosphate cycle photosynthesis, light-harvesting in photosynthesis, pigment binding, chloroplast envelope, and photosynthesis (Table S6) were highly represented among the significantly regulated genes in AL. Additionally, the result showed that many of the genes related to photosynthesis were transcriptionally downregulated in AL.

Chlorophyll metabolism, including chlorophyll biosynthesis, chlorophyll cycling, and chlorophyll degradation, is a complex biological process in plants. Twelve genes engaging 10 enzymes exhibited significant regulation in AL. One of the key factors was that the content of chlorophyll was much lower in AL than in GL. The expression of four chlorophyll biosynthesis genes (encoding HEMA, CHLH, CRD, and POR) was lower in AL than in GL. It has been reported that these enzymes are considered key enzymes for chlorophyll biosynthesis during photomorphogenesis in plants [12,13,14,15,16,17]. Due to the remarkably low levels of expression of these genes, we conclude that chlorophyll biosynthesis activity is lower in AL than in GL. This would explain why the content of chlorophyll a in AL was much lower than in GL. The interconversion of chlorophyll a and chlorophyll b is called the “chlorophyll cycle” [18,19]. Previous studies have reported that a portion of chlorophyll a was converted to chlorophyll b through the activity of CAO. Additionally, chlorophyll b can be reversibly converted to chlorophyll a through 7-hydroxymethyl chlorophyll-a via CBR and 7-hydroxymethyl chlorophyll a reductase (HCAR) [20,21,22]. Two members of *CAO* and three members of *CBR* were downregulated in AL. This might explain why the contents of chlorophyll-a and chlorophyll b in AL were lower than those in GL under the condition of disturbance of chlorophyll a biosynthesis. PAO, which encodes pheophorbide, a oxygenase, catalyzes the oxidation of pheophytin a. Chen et al. reported that the chlorophyll degradation pathway is also called the “PAO pathway” [5]. In our study, compared to GL, two members of PAO expression levels in AL were upregulated, suggesting that chlorophyll degradation was enhanced in ALs of pecan. Based on our results, we hypothesize that the disturbance of chlorophyll a biosynthesis and intensification of chlorophyll degradation lead to the absence of chlorophyll in ALs of pecan.

Abnormal chloroplast structure was observed in yellow and variegated leaves compared with green leaves in *C. sinensis*, and the expression levels of the proteins related to the chlorophyll a-b binding protein, plastid-encoded genes (*Lhcb*, *rbcL*, *rbcS*, *psaA,* and *psbA*), photosystem I P700 chlorophyll A apoprotein A1, photosystem II Qb protein D1, and ribulose bisphosphate carboxylase were remarkably repressed in the variegated leaf, suggesting that the abnormal chloroplast profiles in yellow leaf and variegated leaf might be connected with the downregulation of the abovementioned proteins in *C. sinensis* [1]. The transcripts of differentially expressed proteins related to PSI subunits, PSII subunits, antenna proteins, cytochrome b6/f complex, and beta F-type ATPase were declined in yellow and variegated leaves compared with green leaves in *C. sinensis* [1]. Thus, a dramatic downregulation of proteins related to the photosystem might be linked to abnormal chloroplast profiles. In this study, most of the genes related to the PSI subunits, PSII subunits, cytochrome b6/f complex, photosynthetic electron transcript, F-type ATPase, and photosynthesis-antenna proteins were declined significantly in AL comparing with GL (Table 5), declaring that the photosynthetic system embedded within the thylakoid membrane of the chloroplast was disturbed in ALs of pecan.

Most of the transcript factors play important roles in developmental processes in plants [23]. In tomato fruit, SlMYB72 directly targets protochlorophyllide reductase, Mg-chelatase H subunit, and knotted1-like homeobox2 genes and regulates chlorophyll biosynthesis and chloroplast development [24]. Kiwifruit MYB7 plays a role in modulating carotenoid and chlorophyll pigment accumulation in tissues through transcriptional activation of metabolic pathway genes [25]. LfWRKY70, LfWRKY75, LfWRKY65, LfNAC1, LfSPL14, LfNAC100, and LfMYB113 were shown to be key regulators of leaf senescence, and the genes regulated by LfWRKY75, LfNAC1, and LfMYB113 are candidates to link chlorophyll degradation and anthocyanin biosynthesis to senescence in *Formosan gum* [26]. The LHCB members, which are the apoproteins of the light-harvesting complex of photosystem II, were shown to be targets of WRKY40. Additionally, the positive function of LHCBs was balanced through WRKY40 by repressing the expression of LHCB in ABA signaling [27]. The overexpression of *SlNAC1* resulted in reduced carotenoids by altering carotenoid pathway flux and decreasing ethylene synthesis, mediated mainly by the reduced expression of ethylene biosynthetic genes of system-2 in tomato [28]. Reduced expression of SlNAC4 by RNA interference (*RNAi*) in tomato resulted in delayed fruit ripening, suppressed chlorophyll breakdown, and decreased ethylene synthesis [29]. Plenty of differentially expressed transcript factor members were identified in this study, including MYB, NAC, and WRKY (Table 6), indicating that those transcript factor members were involved in leaf formation in pecan.

## 4. Materials and Methods

### 4.1. Plant Materials and Sample Preparation

For this study, the mutant material with albino leaves was found in a nursery during the seedings of pecan (Figure S1). The seedlings were planted in seedbeds at the Institute of Botany, Jiangsu Province, and the Chinese Academy of Sciences, Jiangsu, China. The substrate contained peat, perlite, and vermiculite in the ratio 5:1:1. The growth conditions consisted of relative humidity of ~60%, a 12 h light/12 h dark photoperiod for 24 h, and a mean temperature of 25 °C. The albino leaves (ALs) and green leaves (GLs) were harvested from six-month-old seedlings. Three independent biological replicates were performed, and each replicate was collected from a pecan seedling. All samples were flash-frozen in liquid nitrogen and stored at −80 °C for future experiments.

### 4.2. Chlorophyll and Carotenoid Content Analysis

Chlorophyll and carotenoid contents were measured using high-performance liquid chromatography (HPLC), as published by Montefiori et al. [30]. Samples were ground into powder in liquid nitrogen and extracted with acetone. Chlorophyll and carotenoid contents were analyzed in biological triplicate.

### 4.3. RNA Isolation, cDNA Library Preparation and Sequencing

Total RNA was extracted from roots using the cetyltrimethylammonium bromide(CTAB) method [31] and then concentrated using oligo (dT) magnetic adsorption. The cDNA library was constructed using an Illumina TruSeq RNA Sample Preparation Kit (Illumina, San Diego, CA, United States). The samples were sequenced using the Illumina HiSeq 2000 machine in Nanjing Genepioneer Biotechnologies Co Ltd., China.

### 4.4. Analysis of Differentially Expressed Genes

After adaptor trimming and quality trimming, the clean reads were mapped to the pecan (Carya illinoensis) transcriptome (Cil.genome.fa, ftp://parrot.genomics.cn/gigadb/pub/10.5524/100001_101000/100571/) using HISAT2. The RPKM (reads per kilobase of exon model per million mapped reads) [32] values were preferred in order to measure the expression of reads using the software StringTie (The Center for Computational Biology at Johns Hopkins University, Baltimore, Maryland, United States). Gene expression differences between log 2 and early stationary phases were obtained by DESeq2 software (European Molecular Biology Laboratory, Heidelberg, Germany) [33]. We defined genes with at least 2-fold change between two samples and FDR (false discovery rate) less than 0.05 as differentially expressed genes. All differentially expressed gene sequences were searched against GenBank’s nonredundant (nr) protein, Swiss-Prot, KEGG, and COG databases using BLASTx to identify the most descriptive annotation for each sequence. In order to understand the biological functions of genes, gene ontology (GO) enrichment (*p*-value < 0.05) was studied by exposing all DEGs to the GO database (http://www.geneontology.org/) to further classify genes or their products into terms (molecular function, biological process, and cellular component). Pathway projects were performed according to the KEGG pathway database in order to perform pathway enrichment analysis of DEGs.

### 4.5. Illumina RNA-seq Result Validation by qRT-PCR

To validate the Illumina RNA-seq results, the differentially expressed genes related to chlorophyll metabolism were selected for qRT-PCR analysis. RNA was isolated from leaves using the abovementioned methods [31], and RNA quality and quantity met the requirements of the qRT-PCR experiment. First-strand cDNA synthesis was performed using the PrimeScript RT Reagent Kit with gDNA Eraser (Takara, Dalian, China) according to the manufacturer’s protocol. The primer sequences used were designed based on gene sequences and Beacon designer software (PREMIER Biosoft, San Francisco, CA, USA), as shown in Table S9 in this study. To ensure gene-specific amplification, normal PCR reactions were performed with the primers (Table S9) to amplify the target genes. A single PCR fragment of the expected size was amplified, suggesting that the primers were suitable for qRT-PCR analyses. The resulting PCR products were cloned and sequenced to confirm the expected fragment of the target genes. qRT-PCR was carried out, as previously described [34], on an Applied Biosystems 7300 Real-Time PCR System (Applied Biosystems, Waltham, MA, USA) using TaKaRa Company SYBR Premix Ex TaqTM II (Perfect Real Time, TaKaRa, code: DRR041A, Dalian, China). Dissociation curves from 55 to 95 °C were generated for each reaction to ensure specific amplification. The *CiActin* gene was used as a positive internal control [35]. The relative levels of genes to control actin mRNAs were analyzed using the 7300 System’s software (Applied Biosystems, Waltham, MA, USA) and the 2^−DDCt^ method [36].

## 5. Conclusions

A total of 5171 DEGs was identified in the comparison of AL vs. GL through de novo transcriptome sequencing; 2216 DEGs (42.85%) were upregulated and 2955 DEGs (57.15%) were downregulated. Chlorophyll contents in AL were significantly lower than those in GL. Additionally, the expression of genes related to chlorophyll biosynthesis (*HEMA1*, *ChlH*, *CRD*, and *POR*) in AL was significantly suppressed and chlorophyll degradation (*PAO*) genes were enhanced in AL, suggesting that the disturbance of chlorophyll biosynthesis and the intensification of chlorophyll degradation lead to the absence of chlorophyll in ALs of pecan. Genes associated with the chloroplast thylakoid membrane, photosystem I, photosystem II, chlorophyll binding, reductive pentose–phosphate cycle photosynthesis, light-harvesting in photosynthesis, pigment binding, chloroplast envelope, and photosynthesis were highly represented in AL, indicating that photosynthesis was destroyed in ALs. Plenty of genes associated with photosynthesis were regulated in AL, declaring that the photosynthetic system embedded within the thylakoid membrane of chloroplast was disturbed in ALs of pecan. These results indicated that the photosynthetic system disturbance was the key cause for the formation of an albino leaf phenotype in pecan. This study provides the theoretical basis for elucidating the molecular mechanism underlying the phenotype of chlorina seedlings of pecan.

## Figures and Tables

**Table 1:** Sequencing and statistics for the two group’s transcriptome data with the reference genome (Cil.genome.fa).

Group Name	GL	AL
No. of total reads (×10^6^)	38.51 ± 5.36	38.68 ± 4.23
No. of mapped reads (×10^6^)	35.87 ± 4.90	35.78 ± 4.08
Mapped percentage	93.15% ± 0.27%	92.46% ± 0.47%
Unique Mapped reads (×10^6^)	34.93 ± 3.87	34.90 ± 3.21

**Table 2:** Summary of functional annotation of the differentially expressed genes.

Database	37, 254 All Unigenes		5171 Differentially Expressed Genes	
	Number of Annotated Sequences	Percentage of Annotated Sequences	Number of Annotated Sequences	Percentage of Annotated Sequences
NR (E-value < 10^−5^)	33,859	90.89	4389	84.88
SwissProt	26,558	71.29	3596	69.54
COG	11,789	31.64	1546	29.90
KEGG	7701	20.67	939	18.16
GO	24,670	66.22	3337	64.53

**Table 3:** The top-10 KEGG pathways mapping enriched differential progress.

Pathway	Pathway ID	Q-Value	DEGs
Photosynthesis	ko00195	1.86 × 10^−24^	51
Photosynthesis-antenna proteins	ko00196	2.86 × 10^−14^	22
Metabolic pathways	ko01100	7.84 × 10^−11^	360
Nitrogen metabolism	ko00910	1.66 × 10^−7^	32
Biosynthesis of secondary metabolites	ko01110	1.70 × 10^−6^	182
Carbon fixation in photosynthetic organisms	ko00710	2.41 × 10^−6^	35
Starch and sucrose metabolism	ko00500	1.01 × 10^−5^	57
Glyoxylate and dicarboxylate metabolism	ko00630	0.0008132	24
Alanine, aspartate and glutamate metabolism	ko00250	0.0008106	22
Glycine, serine and threonine metabolism	ko00260	0.0008446	25

**Table 4:** Identified differentially expressed proteins involved in chlorophyll metabolism.

Genes ID	Protein	Gene	log2(AL/GL)	*p*-Value	Regulated
CIL0203S0025	PREDICTED: glutamyl-tRNA reductase 1, chloroplastic-like	*HEMA*	−1.16362526	1.22 × 10^−7^	down
CIL1034S0072	PREDICTED: magnesium-chelatase subunit ChlH, chloroplastic	*CHLH*	−2.02856009	5.50 × 10^−24^	down
CIL1166S0028	PREDICTED: magnesium-protoporphyrin (oxidative) cyclase, chloroplastic	*CRD*	−2.02856009	5.50 × 10^−24^	down
CIL1444S0041	PREDICTED: chloroplastic	*POR*	−1.85284122	1.14 × 10^−19^	down
CIL1192S0055	PREDICTED: chlorophyllide a oxygenase, chloroplastic isoform ×1	*CAO*	−1.97608744	4.99 × 10^−43^	down
CIL1335S0038	PREDICTED: chlorophyllide a oxygenase, chloroplastic-like	*CAO*	−2.03958666	1.67 × 10^−16^	down
CIL0897S0166	PREDICTED: probable chlorophyll(ide) b reductase NYC1, chloroplastic	*CBR*	−1.31052847	1.92 × 10^−21^	down
CIL1224S0038	PREDICTED: probable chlorophyll(ide) b reductase NYC1, chloroplastic	*CBR*	−1.21082238	1.61 × 10^−15^	down
CIL0897S0167	PREDICTED: probable chlorophyll(ide) b reductase NYC1, chloroplastic	*CBR*	−1.31052847	1.92 × 10^−21^	down
CIL1230S0045	PREDICTED: protein STAY-GREEN LIKE, chloroplastic-like	*SGR*	−1.67002437	1.22 × 10^−13^	down
CIL0946S0047	PREDICTED: pheophorbide a oxygenase, chloroplastic-like	*PAO*	1.48825352	2.60 × 10^−58^	up
CIL1523S0003	PREDICTED: pheophorbide a oxygenase, chloroplastic-like	*PAO*	1.232943538	1.36 × 10^−8^	up

**Table 5:** Identified differentially expressed genes involved in the photosynthesis pathway.

Genes ID	Protein	Gene	log2 (AL/GL)	*p*-Value	Regulated
**Photosystem I**					
MSTRG.12608	Photosystem I P700 apoprotein A1 (chloroplast)	*PsaA*	−1.70701	1.18 × 10^−9^	down
MSTRG.1756	Photosystem I iron-sulfur center	*PsaC*	1.571126	5.12 × 10^−13^	up
CIL1061S0088	PREDICTED: photosystem I reaction center subunit II, chloroplastic-like	*PsaD*	−2.06416	6.02 × 10^−46^	down
CIL1130S0012	PREDICTED: photosystem I reaction center subunit IV, chloroplastic-like	*PsaE*	−2.64087	1.29 × 10^−66^	down
CIL1293S0081	PREDICTED: photosystem I reaction center subunit IV B, chloroplastic-like	*PsaE2*	−2.24447	1.54 × 10^−28^	down
CIL0957S0008	PREDICTED: photosystem I reaction center subunit III, chloroplastic-like	*PsaF*	−2.27227	4.74 × 10^−43^	down
CIL1454S0024	PREDICTED: photosystem I reaction center subunit V, chloroplastic	*PsaG*	−2.15252	1.79 × 10^−45^	down
CIL0121S0006	PREDICTED: photosystem I reaction center subunit VI, chloroplastic-like	*PsaH*	−3.31839	1.62 × 10^−68^	down
CIL1184S0026	PREDICTED: photosystem I reaction center subunit VI-2, chloroplastic	*PsaH2*	−1.80355	9.55 × 10^−31^	down
CIL0225S0008	PREDICTED: photosystem I reaction center subunit psaK, chloroplastic-like	*PsaK*	−2.88506	7.68 × 10^−70^	down
CIL1120S0015	PREDICTED: photosystem I reaction center subunit psaK, chloroplastic-like	*PsaK*	−2.10015	0.000806	down
CIL0070S0007	PREDICTED: photosystem I reaction center subunit XI, chloroplastic	*PsaL*	−2.34772	8.07 × 10^−41^	down
CIL0479S0005	PREDICTED: photosystem I reaction center subunit N, chloroplastic-like	*PsaN*	−2.98776	1.44 × 10^−13^	down
CIL1492S0029	PREDICTED: photosystem I reaction center subunit N, chloroplastic	*PsaN*	−2.11471	2.07 × 10^−42^	down
CIL0899S0004	PREDICTED: photosystem I subunit O	*PsaO*	−2.38235	8.89 × 10^−52^	down
**Photosystem II**					
CIL0840S0001	Putative photosystem II protein D1(Helianthus annuus)	*PsbA*	−3.25158	9.61 × 10^−43^	down
MSTRG.6696	Photosystem II CP47 chlorophyll apoprotein, partial (plastid)	*PsbB*	−2.09893	0.001337	down
MSTRG.28118	PsbB, partial (chloroplast)	*PsbB*	−1.90447	0.000545	down
MSTRG.12382	Photosystem II protein K (chloroplast)	*psbK*	−2.14062	5.63 × 10^−16^	down
MSTRG.11850	Photosystem II protein K (chloroplast)	*psbK*	−2.0512	4.08 × 10^−29^	down
CIL1409S0029	PREDICTED: oxygen-evolving enhancer protein 1, chloroplastic-like	*PsbO*	−3.25073	1.79 × 10^−72^	down
CIL0990S0108	PREDICTED: psbP-like protein 1, chloroplastic	*PsbP*	−1.03085	9.74 × 10^−12^	down
CIL0990S0110	PREDICTED: photosynthetic NDH subunit of lumenal location 1, chloroplastic	*PsbP*	−1.82176	6.22 × 10^−33^	down
CIL1351S0018	PREDICTED: oxygen-evolving enhancer protein 2, chloroplastic	*PsbP*	−1.81357	1.90 × 10^−31^	down
CIL1099S0039	PREDICTED: oxygen-evolving enhancer protein 3-2, chloroplastic-like	*PsbQ*	−2.36913	7.49 × 10^−56^	down
CIL1258S0023	PREDICTED: photosynthetic NDH subunit of lumenal location 2, chloroplastic	*PsbQ*	−2.24784	9.86 × 10^−43^	down
CIL1577S0034	PREDICTED: photosynthetic NDH subunit of lumenal location 3, chloroplastic	*PsbQ*	−2.0615	1.01 × 10^−36^	down
CIL1048S0062	PREDICTED: photosystem II 10 kDa polypeptide, chloroplastic	*PsbR*	−1.39211	3.63 × 10^−21^	down
CIL1112S0006	PREDICTED: photosystem II 22 kDa protein, chloroplastic	*PsbS*	−1.62697	6.31 × 10^−13^	down
CIL1192S0058	PREDICTED: photosystem II 22 kDa protein, chloroplastic	*PsbS*	−1.7504	6.05 × 10^−43^	down
CIL0970S0081	PREDICTED: photosystem II reaction center W protein, chloroplastic-like	*PsbW*	−2.1531	1.81 × 10^−36^	down
CIL1034S0038	PREDICTED: photosystem II reaction center W protein, chloroplastic-like isoform X1	*PsbW*	−1.00199	4.32 × 10^−5^	down
CIL0929S0055	PREDICTED: photosystem II core complex proteins psbY, chloroplastic	*PsbY*	−2.07095	1.48 × 10^−28^	down
CIL1040S0004	PREDICTED: photosystem II repair protein PSB27-H1, chloroplastic	*PSB27-H1*	−2.45399	7.91 × 10^−65^	down
**Cytochrome b6-f complex**					
MSTRG.12087	Cytochrome b6 (chloroplast)	*petB*	−1.12581	7.22 × 10^−5^	down
CIL1135S0039	PREDICTED: cytochrome b6-f complex iron-sulfur subunit 1, chloroplastic-like	*petC*	−1.62437	1.20 × 10^−24^	down
CIL1405S0081	PREDICTED: cytochrome b6-f complex iron-sulfur subunit 1, chloroplastic-like	*petC*	−1.64744	2.11 × 10^−47^	down
**Photosynthetic electron transport**					
CIL0131S0022	Plastocyanin	*petE*	−3.7498	9.31 × 10^−59^	down
CIL1192S0070	PREDICTED: plastocyanin B&apos;/B&apos;&apos	*petE*	−2.19915	7.47 × 10^−48^	down
CIL1082S0115	PREDICTED: ferredoxin-like	*petF*	−1.92418	1.88 × 10^−68^	down
CIL1146S0002	ferredoxin--NADP reductase, leaf-type isozyme, chloroplastic	*petH*	−1.84692	6.72 × 10^−27^	down
CIL1219S0022	PREDICTED: ferredoxin--NADP reductase, root isozyme, chloroplastic	*petH*	1.558778	2.18 × 10^−11^	up
CIL1099S0062	PREDICTED: cytochrome c6, chloroplastic-like	*petJ*	−1.07258	1.22 × 10^−7^	down
**F-Type ATPase**					
CIL0009S0016	ATP synthase beta subunit, partial (chloroplast)	*atpB*	−2.28295	1.64 × 10^−10^	down
CIL0922S0023	PREDICTED: ATP synthase gamma chain, chloroplastic	*atpC*	−1.31948	1.26 × 10^−32^	down
CIL1157S0078	PREDICTED: ATP synthase delta chain, chloroplastic-like	*atpD*	−2.8914	8.90 × 10^−61^	down
CIL1064S0097	PREDICTED: ATP synthase subunit delta, chloroplastic-like	*atpH*	−1.74555	4.63 × 10^−24^	down
CIL0936S0006	ATP synthase F0, A subunit	*A*	1.238679	3.76 × 10^−5^	up
CIL1413S0005	REDICTED: ATP synthase subunit b&apos; chloroplastic	*B*	−1.73514	7.32 × 10^−46^	down
MSTRG.3669	ATPase subunit 8 (mitochondrion)	*B*	1.377949	0.007137	up
**Photosynthesis—antenna proteins**					
CIL1196S0069	PREDICTED: chlorophyll a-b binding protein 6A, chloroplastic-like	*LHCA1*	−3.0003	1.28 × 10^−57^	down
CIL1348S0032	PREDICTED: chlorophyll a-b binding protein 6, chloroplastic-like	*LHCA1*	−3.25903	1.92 × 10^−53^	down
CIL1458S0020	Chlorophyll a-b binding protein, chloroplastic	*LHCA2*	−2.35787	3.49 × 10^−50^	down
CIL1486S0034	PREDICTED: chlorophyll a-b binding protein, chloroplastic	*LHCA2*	−2.93568	1.46 × 10^−48^	down
CIL1118S0087	PREDICTED: chlorophyll a-b binding protein 8, chloroplastic	*LHCA3*	−2.10753	3.33 × 10^−45^	down
CIL1578S0015	Chlorophyll a-b binding protein 4, chloroplastic	*LHCA4*	−3.58717	1.06 × 10^−73^	down
CIL1507S0004	PREDICTED: photosystem I chlorophyll a/b-binding protein 5, chloroplastic	*LHCA5*	−1.20413	1.03 × 10^−14^	down
CIL1582S0038	PREDICTED: photosystem I chlorophyll a/b-binding protein 6, chloroplastic	*LHCA6*	−2.17269	8.46 × 10^−33^	down
CIL0258S0015	Chlorophyll a-b binding protein of LHCII type 1	*LHCB1*	−2.83684	3.34 × 10^−44^	down
CIL1047S0026	Chlorophyll a-b binding protein of LHCII type 1	*LHCB1*	−3.63831	9.87 × 10^−74^	down
CIL1187S0057	Chlorophyll a-b binding protein of LHCII type 1	*LHCB1*	−2.82388	1.67 × 10^−29^	down
CIL1393S0038	Chlorophyll a-b binding protein of LHCII type 1	*LHCB1*	−4.16137	5.33 × 10^−123^	down
CIL1384S0010	PREDICTED: chlorophyll a-b binding protein 151, chloroplastic	*LHCB2*	−3.65164	3.05 × 10^−102^	down
CIL1078S0110	PREDICTED: chlorophyll a-b binding protein 13, chloroplastic	*LHCB3*	−4.01404	5.76 × 10^−57^	down
CIL1135S0055	PREDICTED: chlorophyll a-b binding protein 13, chloroplastic	*LHCB3*	−6.02437	4.65 × 10^−51^	down
CIL1122S0046	PREDICTED: chlorophyll a-b binding protein CP29.1, chloroplastic-like	*LHCB4*	−2.90723	5.76 × 10^−59^	down
CIL1204S0057	PREDICTED: chlorophyll a-b binding protein CP29.3, chloroplastic	*LHCB4*	−3.62946	1.16 × 10^−51^	down
CIL0010S0054	PREDICTED: chlorophyll a-b binding protein CP26, chloroplastic-like	*LHCB5*	−2.54986	7.61 × 10^−42^	down
CIL0948S0124	PREDICTED: chlorophyll a-b binding protein CP26, chloroplastic	*LHCB5*	−1.60257	0.000693	down
CIL0948S0127	PREDICTED: chlorophyll a-b binding protein CP26, chloroplastic	*LHCB5*	−2.17918	5.06 × 10^−35^	down
CIL0424S0004	PREDICTED: chlorophyll a-b binding protein CP24 10A, chloroplastic	*LHCB6*	−2.83352	1.64 × 10^−41^	down
CIL1082S0041	PREDICTED: chlorophyll a-b binding protein CP24 10A, chloroplastic-like	*LHCB6*	−3.78467	7.49 × 10^−69^	down

**Table 6:** Response of transcription factors in the comparison of AL vs. GL.

Category	Total	Upregulation	Downregulation
MYB	277	16	24
AP2/ERF	197	23	12
NAC	122	25	8
C2C2	110	6	16
C2H2	149	14	6
bHLH	159	8	11
WRKY	93	7	12
HB	112	9	4
bZIP	81	7	5
GARP	63	3	5
LOB	54	4	4
B3	69	3	4
MADS	63	2	5
AUX/IAA	43	1	5
GRF	14	6	0

## Supplementary Materials

The following are available online at https://www.mdpi.com/1422-0067/21/17/6137/s1, Figure S1: Albino leaf seedlings of pecan. Figure S2: The correlation coefficient in the repeat group of ALs and GLs. Figure S3: QRT-PCR analysis validation, Table S1: Content of chlorophyll and carotenoid in leaves of green leaves (GL) seedling and albino leaves (AL) seedling in pecan, Table S2: Information statistics of data after filtering and mapped to the pecan genome. Table S3: List of differentially expressed genes (DEGs) in albino leaf compared to green leaf. Table S4: Function annotation of DEGs. Table S5: Gene ontology (GO) functional annotation of DEGs. Table S6: GO function of DEGs. Table S7: KEGG pathway mapping. Table S8: Transcription factors differentially expressed in the comparison of AL vs GL. Table S9: Genes primers in this paper.
